# Supplementary material for: Parental experiences of a diagnosis of neonatal diabetes and perceptions of newborn screening for glucose: a qualitative study
Source: BMJ Open. 2020 Nov 4;10(11):e037312. doi: 10.1136/bmjopen-2020-037312 (PMC7643500; doi:10.1136/bmjopen-2020-037312)
Supplement: Supplementary data [file bmjopen-2020-037312supp001.pdf]

Newbie study: Interview schedule:

Introduction
Re-confirmation of agreement to interview / audio-recording etc

Explanation of purpose of interview: to gain understanding of their experiences and views (no 'right or wrong' answers, all thoughts welcomed), recognition that may bring back some emotional experiences if there's anything they'd rather not talk about that's fine

Specific topic areas:

- ***Experiences of pregnancy*** - including any awareness of fetal growth problems or known previous experience of genetic insulin production problems
- ***Experiences post-delivery*** - any concerns re baby related to glucose / feeding / growth
- ***How the diabetes was detected*** - lead up to hospital attendance, symptoms, how child presented, age at onset, severity
- ***Experience of diabetes diagnosis*** - reactions to diagnosis, what information was given, when genetic testing was discussed, insulin treatment etc.
- ***Experience / Response to genetic diagnosis -*** how information was explained, what sense they made of this, was this linked to information about treatment change, was there discussion about potential learning difficulties, were they put in touch with other families, what support was provided
- ***Did their child have a heel prick at day 5 of life? -*** What was their understanding / experience of this test, how did they feel about it?
- ***What do they think about a test for glucose being added to that test?*** - can they see any advantages or disadvantages of glucose being tested at day 5, would it have made a difference to them and if so how, what do they feel about glucose being added to that test
- ***What are their experiences since the genetic diagnosis?*** *How is their child doing now, general progress, diabetes treatment, hospital follow*
- ***Anything else they may feel is important.*** *Any topics they'd like to discuss or raise that hasn't been asked about specifically, anything else they'd like to provide further detail of, any advice for healthcare professionals or other families*

General prompts (to be used as needed)

- Could you tell me more about that ?
- How did that make you feel ?
- What did you think about that ?

Close of interview,

Check how they are feeling having talked through their experience.

Explanation that draft paper will be sent on to them for comments, any additional thoughts or questions to get in touch, thank them for their time and involvement
