# Supplementary material for: Parental experiences of a diagnosis of neonatal diabetes and perceptions of newborn screening for glucose: a qualitative study
Source: BMJ Open. 2020 Nov 4;10(11):e037312. doi: 10.1136/bmjopen-2020-037312 (PMC7643500; doi:10.1136/bmjopen-2020-037312)
Supplement: Author's manuscript [file bmjopen-2020-037312.draft_revisions.pdf]

BMJ Open

BMJ Open is committed to open peer review. As part of this commitment we make the peer review history of every article we publish publicly available.

When an article is published we post the peer reviewers' comments and the authors' responses online. We also post the versions of the paper that were used during peer review. These are the versions that the peer review comments apply to.

The versions of the paper that follow are the versions that were submitted during the peer review process. They are not the versions of record or the final published versions. They should not be cited or distributed as the published version of this manuscript.

BMJ Open is an open access journal and the full, final, typeset and author-corrected version of record of the manuscript is available on our site with no access controls, subscription charges or pay-per-view fees (http://bmjopen.bmj.com).

If you have any questions on BMJ Open's open peer review process please email info.bmjopen@bmj.com

BMJ Open

# Parental experiences of a diagnosis of neonatal diabetes and perceptions of new-born screening for glucose: A qualitative study

| | |
|---|---|
| Journal: | *BMJ Open* |
| Manuscript ID | bmjopen-2020-037312 |
| Article Type: | Original research |
| Date Submitted by the Author: | 31-Jan-2020 |
| Complete List of Authors: | Shepherd, Maggie; University of Exeter Medical School, NIHR Clinical Research Facility<br>Knight, Bridget; Royal Devon and Exeter NHS Foundation Trust, Exeter NIHR CRF<br>Laskey, Katherine; Royal Devon and Exeter NHS Foundation Trust, Department of Blood Sciences<br>McDonald, Timothy; Royal Devon and Exeter NHS Foundation Trust, Department of Blood Sciences; University of Exeter, NIHR Exeter Clinical Research Facility |
| Keywords: | DIABETES & ENDOCRINOLOGY, NEONATOLOGY, GENETICS, QUALITATIVE RESEARCH |
| | |

SCHOLARONE™
Manuscripts

*I, the Submitting Author has the right to grant and does grant on behalf of all authors of the Work (as defined in the below author licence), an exclusive licence and/or a non-exclusive licence for contributions from authors who are: i) UK Crown employees; ii) where BMJ has agreed a CC-BY licence shall apply, and/or iii) in accordance with the terms applicable for US Federal Government officers or employees acting as part of their official duties; on a worldwide, perpetual, irrevocable, royalty-free basis to BMJ Publishing Group Ltd ("BMJ") its licensees and where the relevant Journal is co-owned by BMJ to the co-owners of the Journal, to publish the Work in this journal and any other BMJ products and to exploit all rights, as set out in our licence.*

*The Submitting Author accepts and understands that any supply made under these terms is made by BMJ to the Submitting Author unless you are acting as an employee on behalf of your employer or a postgraduate student of an affiliated institution which is paying any applicable article publishing charge ("APC") for Open Access articles. Where the Submitting Author wishes to make the Work available on an Open Access basis (and intends to pay the relevant APC), the terms of reuse of such Open Access shall be governed by a Creative Commons licence – details of these licences and which Creative Commons licence will apply to this Work are set out in our licence referred to above.*

*Other than as permitted in any relevant BMJ Author's Self Archiving Policies, I confirm this Work has not been accepted for publication elsewhere, is not being considered for publication elsewhere and does not duplicate material already published. I confirm all authors consent to publication of this Work and authorise the granting of this licence.*

For peer review only

**Full title: Parental experiences of a diagnosis of neonatal diabetes and perceptions of new-born screening for glucose: A qualitative study**

**Short title:** Parental experience of neonatal diabetes and perception of new-born screening for glucose: A qualitative study

Maggie H Shepherd[1], Bridget A Knight[1], Katherine Laskey[2], Timothy J McDonald[2]

**Affiliations:**

1. Exeter NIHR Clinical Research Facility, Royal Devon and Exeter NHS Foundation Trust / University of Exeter Medical School, Exeter UK
2. Department of Blood Sciences, Royal Devon and Exeter NHS Foundation Trust / University of Exeter Medical School, Exeter UK

**Corresponding author:**

Maggie Shepherd m.h.shepherd@exeter.ac.uk

01392 408261

**Word count**

3160

**Key words**

Neonatal diabetes, New-born screening, genetic testing, qualitative

For peer review only

## Abstract

Neonatal diabetes presents <6 months of life but delays in recognition result in presentation with life-threatening hyperglycaemia/diabetic ketoacidosis. Early identification and rapid genetic diagnosis is crucial and ensures correct treatment/management. Adding 'glucose' to new-born bloodspot screening(NBS) could aid prompt detection but requires evidence of parental acceptance.

### Objectives

Increase understanding of parental experience of presentation/recognition of neonatal diabetes and perceptions of glucose testing within NBS.

### Setting

UK families confirmed with a genetic diagnosis of neonatal diabetes, November 2014-2018, were invited to participate.

### Participants

In-depth qualitative interviews were conducted with 10 parents of 14 children(Table 1). 8 had transient neonatal diabetes: *KCNJ11*(n=5), *ABCC8*(n=1), *6q24*(n=2), 6 had permanent neonatal diabetes: *KCNJ11*(n=4), *INS*(n=1), homozygous *GCK*(n=1).

### Primary and secondary outcome measures

Interviews recorded, transcribed and subjected to thematic content analysis.

### Results

3 key themes emerged:


For peer review only

i) Babies were extremely ill at hospital admission, with extended stays in intensive care required *'She was slipping into a coma. It was horrific, she was so close to death…her sugar was 64..we spent 1 month in intensive care(Participant 1)*

ii) Identification of diabetes was not 'standardised' and perceived a 'chance' finding *'It was a blessing she ended up in ICU..I think if she hadn't gone there it wouldn't have been picked up….I shudder to think what might have happened'(Participant 2)*

iii) Adding glucose to NBS was universally considered extremely positive: *'If you don't catch it early it can lead to permanent brain damage, if that can be prevented..that's a very good initiative'(Participant 3)*

**Conclusions**

Diagnosis of neonatal diabetes is frequently delayed, resulting in critically ill presentation with prolonged intensive care support, immense healthcare costs and extreme familial distress. Potential to detect hyperglycaemia earlier was universally endorsed by parents. This provides the first evidence of acceptability of glucose testing fulfilling Wilson-Jungner criteria for implementation within the NBS programme.

**Strengths and limitations of this study**

- This study sought to gain in-depth parental insight into the presentation and recognition of neonatal diabetes and acceptability of adding glucose to the NBS programme, which is required by national screening bodies to implement this screening test.
- Families were from a variety of ethnic backgrounds and were affected by five different genetic causes of neonatal diabetes, with varied treatments and prognosis.

For peer review only

- Patient and public involvement was central throughout and findings were shared with participants to ensure trustworthiness and credibility of data and analysis.
- Limitations were the small number of families interviewed and therefore findings may not represent the views of all families with neonatal diabetes but data saturation was reached and clear themes identified.

## Introduction

Neonatal Diabetes (NDM) presenting less than 6 months of age is the result of severe insulin deficiency, leading to hyperglycaemia and has a high burden of morbidity and mortality. NDM has an incidence of approximately 1 in 90,000 live births [1-3]. There are 23 known genetic causes which account for >80% of cases of diabetes diagnosed less than 6 months of age. Most patients with NDM present with severe hyperglycaemia and diabetic ketoacidosis, associated with prolonged hospital admission and adverse outcomes including lifelong neurological damage or death [4,5]. In Exeter, the National and International referral and diagnostic centre for neonatal diabetes, we are aware of at least 9 cases of children suffering quadriplegia and brain damage as a result of diabetic ketoacidosis with cerebral oedema and coning requiring lifelong institutional support [5-7]. This morbidity and mortality is likely to be a marked underestimate as brain damage and death from ketoacidosis is often unrecognised or unrecorded. The median duration of diabetes at the time of genetic testing decreased from more than 4 years before 2005 to less than 3 months after 2012 [8]. The prognosis is linked to the severity of the hyperglycaemia and ketoacidosis and crucially, the speed with which the disease is recognised and treatment implemented.



For peer review only - http://bmjopen.bmj.com/site/about/guidelines.xhtml

***Correct genetic diagnosis of NDM is important for effective treatment:***

A correct genetic diagnosis of neonatal diabetes is important as it determines the most effective form of treatment. For the commonest subtypes of neonatal diabetes due to *KCNJ11* or *ABCC8* mutations most individuals (>90%) can be transferred from insulin injections to sulphonylurea tablets with marked improvement in glycaemic control, with reductions in hypoglycaemia and blood glucose monitoring [4, 9-11]. Twenty percent of mutations in these genes cause neurological impairment and improvements in motor and cognitive function have been reported, with the greatest benefits seen with earlier sulphonylurea treatment [6, 12-14], leading to improved glucose control, neurological function and quality of life for the patient and their families [15].

***Potential for earlier diagnosis:***

In the United Kingdom, all neonates are screened at day 5 of life as part of standard routine postnatal care. Sample collection involves a heel prick blood sample collected on specialist blood spot paper cards. These are analysed by one of a network of NHS Neonatal Screening laboratory for nine rare but serious metabolic diseases. This NHS routine screening service offers the potential for early detection of hyperglycaemia, as insulin deficiency from birth results in a hyperglycaemia that can be detected in the first few days of life [15], thereby facilitating earlier treatment and genetic diagnosis of NDM.

For a specific test to be considered viable for NBS a number of criteria are required [16]. Screening for NDM fulfils the majority of these with compelling evidence that early identification would avoid many of the severe complications and mortality



For peer review only - http://bmjopen.bmj.com/site/about/guidelines.xhtml

associated with early unrecognised presentation of hyperglycaemia. However the test should also be considered ‘acceptable’ [16].

Our study aimed to gain understanding of parental experience of the presentation and recognition of neonatal diabetes and their views on the acceptability of adding glucose testing to the national NBS programme.

## Methods

***Recruitment:***

UK families with a child with neonatal diabetes diagnosed from November 2014-November 2018 were recruited to the NEWBIE 1 study (*Establishing the diagnostic accuracy of blood spot glucose to detect hyper/hypoglycaemia at day five of life in patients diagnosed with neonatal diabetes or congenital hyperinsulinism.* Ethical approval was granted by the *South West – Cornwall & Plymouth Research Ethics Committee - NREC no: 18/SW/0070).* As part of this study, a subset of parents, whose child had a confirmed genetic cause of neonatal diabetes, were invited to take part in this qualitative study.

Verbal consent was taken by telephone (by KL) and consent forms were posted to potential participants. Written consent was received through the use of pre-postage paid envelopes and, for those who returned their written consent, interviews were arranged for a time and date that was convenient. As the families had young children and were located across the UK, telephone interviews were considered both convenient and acceptable to parents.

A qualitative approach, from a social constructivist stance, was used to gain in-depth insight into the presentation and recognition of neonatal diabetes, with a focus on



For peer review only - http://bmjopen.bmj.com/site/about/guidelines.xhtml

understanding the meaning these events had for the families being studied, and their views on the acceptability of adding glucose to the NBS programme,

**Patient and public involvement**

Patient and public involvement has been integral throughout the project. The study was initially discussed with 12 members (3 with diabetes) of the NIHR Exeter Clinical Research Facility user group and all agreed the project was feasible and would provide no inconvenience. Three parents of children affected by neonatal diabetes were also contacted and unanimously supported the proposal and two were actively involved in the creating and reviewing patient information. They will also assist us in producing summaries of the project for dissemination to ensure these are clear to non-healthcare professionals and can have maximum impact. The findings of the study have also been shared with the participants as described in the discussion.

In-depth telephone interviews were conducted with 10 parents (8 mothers and 2 fathers) of 14 children with neonatal diabetes, currently aged 1-14 years, median 2 yrs. Eight of the children had transient neonatal diabetes (*KCNJ11* (n=5), *ABCC8* (n=1), *6q24* (n=2) all of whom were in currently remission and 6 had permanent neonatal diabetes: *KCNJ11* (n=4), *INS* (n=1), homozygous *GCK* (n=1). Families were from a variety of ethnic groups: Western European (n=7), Arabic (n=1), Pakistani (n=1), Chinese (n=1) (Table 1).



For peer review only - http://bmjopen.bmj.com/site/about/guidelines.xhtml

175 Table 1. Characteristics of the families taking part

176

| Participant | Gene affected | PNDM / TNDM | Ethnic group | Parent affected | Currently in remission |
|---|---|---|---|---|---|
| 1 | *KCNJ11* | PNDM | Western European | No | N/A |
| 2 | *6q 24* | TNDM | Western European | No | Yes |
| 3 | *KCNJ11* | TNDM | Pakistani | Parents heterozygous (but unaffected, 1st cousins) | Yes |
| 4 | *ABCC8* | TNDM | Western European | Mother | Yes |
| 5 | *GCK* | PNDM | Arabic | Parents heterozygous | N/A |
| 6 | *6q24* | TNDM | Western European | No | Yes |
| 7 | *KCNJ11* | PNDM | Western European | Mother | N/A |
| 8 | *INS* | PNDM | Western European | Mother | N/A |
| 9 | *KCNJ11* | PNDM | Chinese | No | N/A |
| 10 | *KCNJ11* | TDNM | Western European | Mother | Yes |

For peer review only

### *Data Collection and Analysis*

Telephone interviews were conducted with all participants by an experienced qualitative researcher (MS), who introduced herself and her role within this study. She had previously been in contact with four of the parents in her capacity as senior nurse within the monogenic team when providing clinical advice regarding the management of the neonatal diabetes following a genetic diagnosis. Duration of the interviews was determined by the interviewee's responses, but typically lasted 45-60 minutes. Parents were previously asked to identify and remain in, a private, quiet location for the duration of the interview. The interviews were semi-structured with open questions, utilising a qualitative interview guide which focused on six domains:

1. Experiences of pregnancy / post-delivery period
2. Recognition of diabetes
3. Experience of genetic diagnosis
4. Response to diagnosis and experiences since
5. Awareness of NBS tests
6. Views of adding glucose to the NBS and anything else they felt was important to allow issues that were significant to the families to emerge.

The approach taken was flexible and iterative.

The interviews were tape-recorded to ensure accurate data collection and the interviewer also made notes. Interviews were subsequently transcribed verbatim, although names and identifying features were abbreviated to preserve anonymity. All participants were assigned a unique study ID. A thematic content analysis was undertaken using an inductive, manual process to identify key themes and codes and a list of common themes generated. Interviews were concluded after 10



For peer review only - http://bmjopen.bmj.com/site/about/guidelines.xhtml

interviews as data saturation was achieved with no new themes emerging from the data. The transcripts were independently reviewed by another experienced qualitative researcher (BK). The initial data analysis was revisited to refine the themes and develop sub-themes with participant quotes identified which supported the themes. Themes were discussed with the research team and discrepancies resolved by consensus. Recurring patterns were identified and relationships between the themes within and across transcripts were recorded. Anonymised interview transcripts were stored on a password protected computer within a secure (swipe card access only) research environment.

## Results

3 key themes emerged:

### i) ***Babies were extremely ill when admitted to hospital, with long stays in intensive care required***

Although symptoms of hyperglycaemia had been reported by parents prior to admission, these had been dismissed by other healthcare professionals and led to delays in diagnosis

*'They had no suspicion at all that something can be wrong…..even GP, doctors look at her 'Oh yeah she's so good, she's fine' .. One day before* (hospital admission) *I said 'She's drinking a lot of milk ..and she's sick a lot now' and* (they said) *'Oh it can happen it's reflux'* (but) *…she was drinking and drinking and drinking' (Participant 1)*

Other causes of symptoms were assumed and delays in admission resulted

*'She'd not grown since she was 6 weeks old and she kept throwing up her bottles and always demanding more, she was always grumpy ….so we took her to the doctors but there was a sickness bug going round and they said 'Bring her back on Monday' (Participant 4)*



For peer review only - http://bmjopen.bmj.com/site/about/guidelines.xhtml

Many of the babies were considered to be extremely ill when they were finally admitted to hospital and families recognised healthcare professionals concerns and babies were frequently taken straight to the neonatal intensive care units

*'They were very worried, she was taken direct to ICU.. it was a nightmare for me (Participant 5).*

There was clear concern regarding the baby's chances of survival and this impacted emotionally on the families.

*'Stressed, depressed, don't know what was going to happen to him, all a worry… it was frightening and scary and horrible' (Participant 6)*

Consequently long stays of several weeks in intensive care were common

*'He was in neonatal for about 3 weeks' (Participant 7)*

*'He was in hospital for 5 weeks altogether' (Participant 6)*

Delays in recognising the deteriorating health of the neonates and the significance of the symptoms resulted in delays in admission, increased distress for the parents and prolonged hospital stays.

**ii) *Identification of diabetes was not 'standardised' and often perceived to be a 'chance' finding***

A number of the families considered testing of blood glucose was down to 'luck' or the attention of an individual health care professional as opposed to a routinely performed test

For peer review only

*'One of the nurses, she was not asked to do so but, she did a urine sample and they found her sugars were abnormal and that is what made her go into the hospital again so it was lucky for H to find it so early' (Participant 5)*

Where an individual healthcare professional was regarded as being the 'single person' who had identified the problem, questions were raised about what might have transpired if that healthcare professional was not involved

*'It was just this one good doctor who came ..and he was marvellous and luckily he come or I don't know what would have happened ...I mean would B have been in a worse state ? ...maybe he'd have 'gone' for ever (Participant 6)*

One family, where the baby's mother was known to have diabetes herself, had insisted their baby's blood glucose was tested, despite being advised to the contrary

*'She had an abscess above her bottom ..and she was admitted ..* (my) *Dad said 'Mum's diabetic, can you test her* (the baby's) *blood sugars please?' and they tried to fob us off they were 'No, no, no, it won't be anything like that' but he was adamant ..and that's when they did and they* (the blood sugars) *were 18 ... she was 2 weeks* (of age)*... it could have completely got missed' (Participant 8)*

Extremely high levels of glucose on admission were common, suggesting the diagnosis of diabetes had clearly been delayed

*'It was 30s, 36 nearly touching 40 sometimes' (Participant 6)*

*'The blood sugar was extremely high, around 40-50' (Participant 9)*

Healthcare professionals were not perceived to expect diabetes to be present at such an early age and parents highlighted the need for education



For peer review only - http://bmjopen.bmj.com/site/about/guidelines.xhtml

*'They obviously didn't have a clue about this different type of diabetes ..they kept saying his blood sugars were high and they didn't really expect anything like this …they weren't aware … I do really think they need to know more' (Participant 7)*

Healthcare professional's lack of familiarity with neonatal diabetes added to the anxieties felt by the families

*'The most difficult part for me was the beginning, because literally nobody knew what was going on, it wasn't common, nobody knew what to do and it really scared us' (Participant 2)*

Despite a lack of experience with neonatal diabetes, parents praised the care given by the healthcare professionals and recognised this was also a difficult circumstance the teams involved

*'Even though all the medical staff were absolutely amazing I can't fault them …this was a very unknown situation for them… I could see nobody knew what to do really' (Participant 2)*

Parents were hugely appreciative of the care their child received

*'I have been very, very grateful for everything that the diabetes specialist has done for our baby, it actually changed the way I think about the UK health system' (Participant 9)*

Recognition of diabetes and testing of blood glucose was considered by parents to be predominantly a consequence of the location of their baby e.g. in an intensive care setting or down to individual healthcare professionals, as opposed to a routine test undertaken in unwell neonates. Despite healthcare professionals unfamiliarity with neonatal diabetes the families were very grateful for the care their children received.

**iii) *Adding glucose to the NBS test was universally considered extremely positive:***



For peer review only - http://bmjopen.bmj.com/site/about/guidelines.xhtml

Every single parent interviewed was positive about the possibility of adding a glucose test onto the NBS programme

*'I think it's an absolutely fantastic idea I really, really do it's brilliant .. and I think if it wasn't for me carrying the gene ..they wouldn't probably have got tested, so I think it's an absolutely fantastic idea' (Participant 10)*

They recognised this could avoid symptoms of hyperglycaemia being missed

*'I think it's a good idea, that would have been ideal because we went through weeks of .. the symptoms of diabetes but not picking up on it' (Participant 4)*

and recognised that testing at day 5 of life could prevent deterioration in the condition of babies with diabetes

*'That would be a great idea, I mean before it develops even further.. discovering this at a very early stage is going to be extremely helpful' (Participant 9)*

Parents considered this approach would avoid potential problems from a delayed diagnosis

*'She was lucky to be diagnosed at 10 days.. I wonder how she survived. In the future .. it would be much better if they were diagnosed earlier and treated earlier, rather than leaving for later days and leading to damage for the child' (Participant 5)*

They highlighted the possibility of preventing babies becoming seriously ill with all the fear that caused

*'My baby went into coma and nearly died …so if they find it at day 5 …we could have avoided that' (Participant 1)*

Parents were unanimous in the view that adding glucose to the NBS programme was a positive step which would reduce delayed diagnosis and deterioration in the health



For peer review only - http://bmjopen.bmj.com/site/about/guidelines.xhtml

of babies with diabetes and prevent other families from going through the trauma they had as a consequence of a delayed diagnosis.

## Discussion

Our study provides the first evidence of parental support for the addition of glucose to the new-born screening programme and in-depth insights into the experiences around diabetes diagnosis during the neonatal period in five different genetic subtypes. Previous studies have explored attitudes to expanding new-born screening in other conditions but many have focused on the views of clinicians [17] or parents with 'healthy' children [18] with far less attention paid to the views of families living with the conditions [19].

Parental views are recognised to be influenced by contextual factors such as personal experience [19]. In families with haemophilia the vast majority (77%) supported new-born screening predominantly because they considered it a means to facilitate early support and treatment, inform decisions about future pregnancies and prevent the 'diagnostic odyssey' and difficulties associated with a later diagnosis [20]. Concerns about the detrimental impact, in terms of bonding and parental stress, that an unsought and serious diagnosis can have on early parent-child relationships have been highlighted in cystic fibrosis [21,22] and Spinal Muscular Atrophy (SMA)[23]. Despite the majority (70%) of families and individuals living with SMA being in favour of new-born genetic screening due to delays in diagnosis, those not in favour (whose babies had an extremely curtailed lifespan or a longer period of time before the onset of symptoms), were concerned that new-born genetic screening would prevent families enjoying care-free time with their baby before symptoms emerged [23].



For peer review only - http://bmjopen.bmj.com/site/about/guidelines.xhtml

Strengths and Limitations:

A key strength of this study is that participants were from a variety of backgrounds and represented five different genetic causes of neonatal diabetes, with varied treatments and prognosis. Parents were all able to provide 'insider information' on their personal experiences of a delayed diagnosis of neonatal diabetes in their families. Copies of the draft paper were sent to the participants to ensure the trustworthiness and credibility of the data and analysis, with an invitation to provide any feedback to the research team. Although the number of families interviewed was small and therefore our findings may not represent the views of all families with neonatal diabetes, data saturation was reached and clear themes identified.

We have previously shown that glucose levels at day 5 of life in those with neonatal diabetes are already raised and markedly higher than the normal range [15], therefore the introduction of glucose as part of the new-born screening programme could prevent delays in recognition and ensure prompt treatment of neonatal diabetes.

**Conclusion**

Recognition and diagnosis of neonatal diabetes is frequently delayed with babies presenting with extreme hyperglycaemia, requiring extended intensive care support. This leads to high NHS care costs and parental distress. Our study contributes to an emerging literature that considers the acceptability of new-born screening practices from the vantage point of parents with experience of a child with the condition. This study provides the first evidence that adding glucose to the NBS programme is not only acceptable to families but fully endorsed by them. Parents considered the



For peer review only - http://bmjopen.bmj.com/site/about/guidelines.xhtml

addition of a glucose measurement to NBS would reduce delays in the diagnosis of neonatal diabetes and prevent the extent of the suffering they experienced with the deteriorating health of their baby. Our study provides the first evidence of acceptability of testing, fulfilling one of the Wilson-Jungner criteria for adding glucose as part of the NBS programme.

**Acknowledgments**

The NIHR Exeter Clinical Research Facility is a partnership between the University of Exeter Medical School College of Medicine and Health, and Royal Devon and Exeter NHS Foundation Trust. This project is supported by the National Institute for Health Research (NIHR) Exeter Clinical Research Facility. The views expressed are those of the author (s) and not necessarily those of the NIHR or the Department of Health and Social Care.

**Funding**

MS is a National Institute for Health Research (NIHR) Senior Nurse and Midwife Research Leader. MS and BK are supported by the Exeter NIHR Clinical Research Facility at the University of Exeter. TM is funded by an NIHR HEE Senior Clinical Lecturer Fellowship (ICA-SCL-2016-02-003).

**Author contributions:**

MS, BK and TM conceptualised the study and developed the methodology, MS, BK and KL recruited participants. MS conducted and transcribed the interviews, MS and BK analysed the data. MS prepared the original draft, BK, LM and TM reviewed and edited the drafts and MS revised subsequent editions.



For peer review only - http://bmjopen.bmj.com/site/about/guidelines.xhtml

## Conflicts of interest

There are no conflicts of interest to declare

## Data availability statement

Requests for access to data, and to discuss potential collaborations, should be made in writing in the first instance to the Chief Investigator Professor Timothy McDonald timothy.mcdonald@nhs.net

## References:


1. Slingerland AS, Shields BM, Flanagan SE, Bruining GJ, Noordam K, Gach A, et al. Referral rates for diagnostic testing support an incidence of permanent neonatal diabetes in three European countries of at least 1 in 260,000 live births. diabetologia. 2009;52[8]:1683-5.
2. Wiedemann B, Schober E, Waldhoer T, Koehle J, Flanagan SE, Mackay DJ, et al. Incidence of neonatal diabetes in Austria-calculation based on the Austrian Diabetes Register. Pediatr Diabetes. 2010;11[1]:18-23.
3. Iafusco D, Massa O, Pasquino B, Colombo C, Iughetti L, Bizzarri C, et al. Minimal incidence of neonatal/infancy onset diabetes in Italy is 1:90,000 live births. Acta Diabetol. 2012;49[5]:405-8.
4. Pearson ER, Flechtner I, Njolstad PR, Malecki MT, Flanagan SE, Larkin B, et al. Switching from insulin to oral sulfonylureas in patients with diabetes due to Kir6.2 mutations. N Engl J Med. 2006;355[5]:467-77.
5. Gloyn AL, Diatloff-Zito C, Edghill EL, Bellanne-Chantelot C, Nivot S, Coutant R, et al. *KCNJ11* activating mutations are associated with developmental delay, epilepsy and neonatal diabetes syndrome and other neurological features. Eur J Hum Genet. 2006;14[7]:824-30.




For peer review only - http://bmjopen.bmj.com/site/about/guidelines.xhtml

6. Busiah K, Verkarre V, Cave H, Scharfmann R, Polak M. Human pancreas endocrine cell populations and activating *ABCC8* mutations. Horm Res Paediatr. 2014;82[1]:59-64.

7. B Jose UG, T Barrett, AT Hattersley, JJ Milles. Glibenclamide controls ketosis-prone diabetes in a 38-year-old woman with Kir6.2 mutation Practical Diabetes International 2009;26[6]:3.

8. De Franco E, Flanagan SE, Houghton JA, Lango Allen H, Mackay DJ, Temple IK, et al. The effect of early, comprehensive genomic testing on clinical care in neonatal diabetes: an international cohort study. Lancet. 2015;386(9997):957-63.

9. Rafiq M, Flanagan SE, Patch AM, Shields BM, Ellard S, Hattersley AT. Effective treatment with oral sulfonylureas in patients with diabetes due to sulfonylurea receptor 1 (SUR1) mutations. Diabetes Care. 2008;31[2]:204-9.

10. Zung A, Glaser B, Nimri R, Zadik Z. Glibenclamide treatment in permanent neonatal diabetes mellitus due to an activating mutation in Kir6.2. J Clin Endocrinol Metab. 2004;89[11]:5504-7.

11. Clark RH, McTaggart JS, Webster R, Mannikko R, Iberl M, Sim XL, et al. Muscle dysfunction caused by a KATP channel mutation in neonatal diabetes is neuronal in origin. Science. 2010;329(5990):458-61.

12. Slingerland AS, Hurkx W, Noordam K, Flanagan SE, Jukema JW, Meiners LC, et al. Sulphonylurea therapy improves cognition in a patient with the V59M *KCNJ11* mutation. Diabet Med. 2008;25[3]:277-81.

13. Slingerland AS, Nuboer R, Hadders-Algra M, Hattersley AT, Bruining GJ. Improved motor development and good long-term glycaemic control with sulfonylurea treatment in a patient with the syndrome of intermediate developmental delay, early-onset generalised epilepsy and neonatal diabetes



For peer review only - http://bmjopen.bmj.com/site/about/guidelines.xhtml

associated with the V59M mutation in the *KCNJ11* gene. Diabetologia. 2006;49[11]:2559-63.

14. Shah RP, Spruyt K, Kragie BC, Greeley SA, Msall ME. Visuomotor performance in *KCNJ11*- related neonatal diabetes is impaired in children with DEND-associated mutations and may be improved by early treatment with sulfonylureas. Diabetes Care. 2012;35[10]:2086-8.

15. McDonald TJ, Besser RE, Perry M, Babiker T, Knight BA, Shepherd MH, Ellard S, Flanagan SE, Hattersley AT. Screening for neonatal diabetes at day 5 of life using dried blood spot glucose measurement. Diabetologia. 2017 Aug 5. doi: 10.1007/s00125-017-4383-3. [Epub ahead of print)

16. Wilson JM, Jungner YG. [Principles and practice of mass screening for disease]. Bol Oficina Sanit Panam. 1968;65[4]:281-393.

17. Hiraki S, Ormond KE, Kim K, Ross LF. 2006. Attitudes of genetic counselors towards expanding new-born screening and offering predictive genetic testing to children. Am J Med Genet Part A 140A:2312–2319.

18. Hasegawa L.E, Fergus K.A,Ojeda N, Au SM. Parental Attitudes toward Ethical and Social Issues Surrounding the Expansion of New-born Screening Using New Technologies. Public Health Genomics. 2011 Jul; 14(4-5): 298–306.

19. Nicholls SG, Southern KW. Parental Decision-Making and Acceptance of New-born Bloodspot Screening: An Exploratory Study PLoS One. 2013; 8[11]: e79441.



For peer review only - http://bmjopen.bmj.com/site/about/guidelines.xhtml

20. Boardman FK, Hale R, Young PJ. New-born screening for haemophilia: The views of families and adults living with haemophilia in the UK. Haemophilia. 2019 Mar; 25[2]: 276–282.

21. Al-Jader LN, Goodchild MC, Ryley HC, Harper PS. Attitudes of parents of cystic fibrosis children towards neonatal screening and antenatal diagnosis. Clinical genetics. 1990. 38 [6] 460-465

22. Grob R. Is my sick child healthy? Is my healthy child sick?: changing parental experiences of cystic fibrosis in the age of expanded new-born screening. Soc Sci Med. 2008 Oct;67[7]:1056-64.

23. Boardman FK, Young PJ, Griffiths FE. New-born screening for spinal muscular atrophy: The views of affected families and adults. Am J Med Genet A. 2017 Jun; 173[6]: 1546–1561.

For peer review only

# Standards for Reporting Qualitative Research (SRQR)*

http://www.equator-network.org/reporting-guidelines/srqr/

**Page/line no(s).**

## Title and abstract

| | |
|---|---|
| **Title** - Concise description of the nature and topic of the study Identifying the study as qualitative or indicating the approach (e.g., ethnography, grounded theory) or data collection methods (e.g., interview, focus group) is recommended | Page 1 (line 4-5) |
| **Abstract** - Summary of key elements of the study using the abstract format of the intended publication; typically includes background, purpose, methods, results, and conclusions | Page 2-3 (lines 24-63) |

## Introduction

| | |
|---|---|
| **Problem formulation** - Description and significance of the problem/phenomenon studied; review of relevant theory and empirical work; problem statement | Page 3-5 (lines 81-126) |
| **Purpose or research question** - Purpose of the study and specific objectives or questions | Page 6 (lines 128-130) |

## Methods

| | |
|---|---|
| **Qualitative approach and research paradigm** - Qualitative approach (e.g., ethnography, grounded theory, case study, phenomenology, narrative research) and guiding theory if appropriate; identifying the research paradigm (e.g., postpositivist, constructivist/ interpretivist) is also recommended; rationale** | Page 6 (lines 149-152) |
| **Researcher characteristics and reflexivity** - Researchers' characteristics that may influence the research, including personal attributes, qualifications/experience, relationship with participants, assumptions, and/or presuppositions; potential or actual interaction between researchers' characteristics and the research questions, approach, methods, results, and/or transferability | Page 9 (lines 179-183) |
| **Context** - Setting/site and salient contextual factors; rationale** | Page 6 (lines 133-147) and Page 9 (lines 179-186) |
| **Sampling strategy** - How and why research participants, documents, or events were selected; criteria for deciding when no further sampling was necessary (e.g., sampling saturation); rationale** | Page 6 (lines 134-141) Page 9 (lines 196-203) |
| **Ethical issues pertaining to human subjects** - Documentation of approval by an appropriate ethics review board and participant consent, or explanation for lack thereof; other confidentiality and data security issues | Page 6 (lines 133-141) and page 9-10 (line 196-210) |



For peer review only - http://bmjopen.bmj.com/site/about/guidelines.xhtml

| | |
|---|---|
| **Data collection methods** - Types of data collected; details of data collection procedures including (as appropriate) start and stop dates of data collection and analysis, iterative process, triangulation of sources/methods, and modification of procedures in response to evolving study findings; rationale** | Page 7 (line 164-170) Page 9 (line 178-208) |
| **Data collection instruments and technologies** - Description of instruments (e.g., interview guides, questionnaires) and devices (e.g., audio recorders) used for data collection; if/how the instrument(s) changed over the course of the study | Page 9 (lines 179-210) |
| **Units of study** - Number and relevant characteristics of participants, documents, or events included in the study; level of participation (could be reported in results) | Page 7 (line 164-170) Page 8 (table 1) |
| **Data processing** - Methods for processing data prior to and during analysis, including transcription, data entry, data management and security, verification of data integrity, data coding, and anonymization/de-identification of excerpts | Page 9 (line 195-210) |
| **Data analysis** - Process by which inferences, themes, etc., were identified and developed, including the researchers involved in data analysis; usually references a specific paradigm or approach; rationale** | Page 9 (line 196-210) |
| **Techniques to enhance trustworthiness** - Techniques to enhance trustworthiness and credibility of data analysis (e.g., member checking, audit trail, triangulation); rationale** | Page 16 (line 356-360) |

**Results/findings**

| | |
|---|---|
| **Synthesis and interpretation** - Main findings (e.g., interpretations, inferences, and themes); might include development of a theory or model, or integration with prior research or theory | Page 10-15 (line 212-327) |
| **Links to empirical data** - Evidence (e.g., quotes, field notes, text excerpts, photographs) to substantiate analytic findings | Page 10-14 (line 212-323) |

**Discussion**

| | |
|---|---|
| **Integration with prior work, implications, transferability, and contribution(s) to the field -** Short summary of main findings; explanation of how findings and conclusions connect to, support, elaborate on, or challenge conclusions of earlier scholarship; discussion of scope of application/generalizability; identification of unique contribution(s) to scholarship in a discipline or field | Page 15 (lines 330-336) |
| **Limitations** - Trustworthiness and limitations of findings | Page 16 (lines 354-362) Page 3 (line 66-78) |

**Other**

| | |
|---|---|
| **Conflicts of interest** - Potential sources of influence or perceived influence on study conduct and conclusions; how these were managed | Page 18 (line 405) |



For peer review only - http://bmjopen.bmj.com/site/about/guidelines.xhtml

| | |
|---|---|
| **Funding** - Sources of funding and other support; role of funders in data collection, interpretation, and reporting | Page 17 (lines 393-396) |

*The authors created the SRQR by searching the literature to identify guidelines, reporting standards, and critical appraisal criteria for qualitative research; reviewing the reference lists of retrieved sources; and contacting experts to gain feedback. The SRQR aims to improve the transparency of all aspects of qualitative research by providing clear standards for reporting qualitative research.

**The rationale should briefly discuss the justification for choosing that theory, approach, method, or technique rather than other options available, the assumptions and limitations implicit in those choices, and how those choices influence study conclusions and transferability. As appropriate, the rationale for several items might be discussed together.

For peer review only

**Reference:**

O'Brien BC, Harris IB, Beckman TJ, Reed DA, Cook DA. **Standards for reporting qualitative research: a synthesis of recommendations.** *Academic Medicine*, Vol. 89, No. 9 / Sept 2014 DOI: 10.1097/ACM.0000000000000388

BMJ Open

# Parental experiences of a diagnosis of neonatal diabetes and perceptions of new-born screening for glucose: A qualitative study



| | |
|---|---|
| Journal: | *BMJ Open* |
| Manuscript ID | bmjopen-2020-037312.R1 |
| Article Type: | Original research |
| Date Submitted by the Author: | 06-Jul-2020 |
| Complete List of Authors: | Shepherd, Maggie; University of Exeter, Institute of Biomedical and Clinical Science / NIHR Clinical Research Facility<br>Knight, Bridget; Royal Devon and Exeter NHS Foundation Trust, Exeter NIHR CRF<br>Laskey, Katherine; Royal Devon and Exeter NHS Foundation Trust, Department of Blood Sciences<br>McDonald, Timothy; Royal Devon and Exeter NHS Foundation Trust, Department of Blood Sciences; University of Exeter, NIHR Exeter Clinical Research Facility |
| <b>Primary Subject Heading</b>: | Diabetes and endocrinology |
| Secondary Subject Heading: | Genetics and genomics, Qualitative research, Diagnostics |
| Keywords: | DIABETES & ENDOCRINOLOGY, NEONATOLOGY, GENETICS, QUALITATIVE RESEARCH |
| | |



SCHOLARONE™
Manuscripts

*I, the Submitting Author has the right to grant and does grant on behalf of all authors of the Work (as defined in the below author licence), an exclusive licence and/or a non-exclusive licence for contributions from authors who are: i) UK Crown employees; ii) where BMJ has agreed a CC-BY licence shall apply, and/or iii) in accordance with the terms applicable for US Federal Government officers or employees acting as part of their official duties; on a worldwide, perpetual, irrevocable, royalty-free basis to BMJ Publishing Group Ltd ("BMJ") its licensees and where the relevant Journal is co-owned by BMJ to the co-owners of the Journal, to publish the Work in this journal and any other BMJ products and to exploit all rights, as set out in our licence.*

*The Submitting Author accepts and understands that any supply made under these terms is made by BMJ to the Submitting Author unless you are acting as an employee on behalf of your employer or a postgraduate student of an affiliated institution which is paying any applicable article publishing charge ("APC") for Open Access articles. Where the Submitting Author wishes to make the Work available on an Open Access basis (and intends to pay the relevant APC), the terms of reuse of such Open Access shall be governed by a Creative Commons licence – details of these licences and which Creative Commons licence will apply to this Work are set out in our licence referred to above.*

*Other than as permitted in any relevant BMJ Author's Self Archiving Policies, I confirm this Work has not been accepted for publication elsewhere, is not being considered for publication elsewhere and does not duplicate material already published. I confirm all authors consent to publication of this Work and authorise the granting of this licence.*

For peer review only

**Full title: Parental experiences of a diagnosis of neonatal diabetes and perceptions of new-born screening for glucose: A qualitative study**

**Short title:** Parent's perception of new-born screening for neonatal diabetes: A qualitative study

Maggie H Shepherd[1], Bridget A Knight[1], Katherine Laskey[2], Timothy J McDonald[2]

**Affiliations:**

1. Exeter NIHR Clinical Research Facility, Royal Devon and Exeter NHS Foundation Trust / University of Exeter Medical School, Exeter UK
2. Department of Blood Sciences, Royal Devon and Exeter NHS Foundation Trust / University of Exeter Medical School, Exeter UK

**Corresponding author:**

Maggie Shepherd m.h.shepherd@exeter.ac.uk

01392 408261

**Word count**

4119

**Key words**

Neonatal diabetes, New-born screening, genetic testing, qualitative



For peer review only - http://bmjopen.bmj.com/site/about/guidelines.xhtml

## Abstract

Neonatal diabetes presents <6 months of life but delays in recognition result in presentation with life-threatening hyperglycaemia/diabetic ketoacidosis. Early identification and rapid genetic diagnosis is crucial and ensures correct treatment/management. Adding 'glucose' to new-born bloodspot screening (NBS) could aid prompt detection but requires evidence of parental acceptance.

### Objectives

Increase understanding of parental experience of presentation/recognition of neonatal diabetes and perceptions of glucose testing within NBS.

### Setting

UK families confirmed with a genetic diagnosis of neonatal diabetes, November 2014-2018, were invited to participate.

### Participants

In-depth qualitative interviews were conducted with 10 parents of 14 children. 8 had transient neonatal diabetes: *KCNJ11* (n=5), *ABCC8* (n=1), *6q24* (n=2), 6 had permanent neonatal diabetes: *KCNJ11* (n=4), *INS* (n=1), homozygous *GCK* (n=1).

### Primary and secondary outcome measures

Interviews audiorecorded, transcribed and subjected to thematic content analysis.

### Results

3 key themes emerged:

i) Babies were extremely ill at hospital admission, with extended stays in intensive care required '


For peer review only

ii) Identification of diabetes was not ‘standardised’ and perceived a ‘chance’ finding

iii) Adding glucose to NBS was universally considered extremely positive:

**Conclusions**

Diagnosis of neonatal diabetes is frequently delayed, resulting in critically ill presentation with prolonged intensive care support, additional healthcare costs and familial distress. Potential to detect hyperglycaemia earlier was universally endorsed by parents with no negative consequences identified. Although further study including a larger number of individuals is needed to confirm our findings this study provides the first evidence of acceptability of glucose testing fulfilling Wilson-Jungner criteria for implementation within the NBS programme,.

**Strengths and limitations of this study**

- This study gained in-depth parental insight into the identification of neonatal diabetes and acceptability of newborn glucose screening.
- Families were from a variety of ethnic backgrounds and affected by five different genetic causes of neonatal diabetes, with varied treatments and prognosis.
- Patient and public involvement was central and findings were shared with participants to ensure trustworthiness and credibility of data and analysis.
- Limitations were the small number of families interviewed and therefore findings may not represent the views of all families with neonatal diabetes.

**Introduction**



For peer review only - http://bmjopen.bmj.com/site/about/guidelines.xhtml

Neonatal Diabetes (NDM) presenting less than 6 months of age is the result of severe insulin deficiency, leading to hyperglycaemia and has a high burden of morbidity and mortality. NDM has an incidence of approximately 1:100,000 but reports range from between 1 in 25,000 to 1:500,000live births [1-4]. There are 23 known genetic causes which account for >80% of cases of diabetes diagnosed less than 6 months of age. Most patients with NDM present with severe hyperglycaemia and diabetic ketoacidosis, associated with prolonged hospital admission and adverse outcomes including lifelong neurological damage or death with children suffering quadriplegia and brain damage as a result of diabetic ketoacidosis with cerebral oedema and coning requiring lifelong institutional support [5-7]. This morbidity and mortality is likely to be a marked underestimate as brain damage and death from ketoacidosis is often unrecognised or unrecorded. The median duration of diabetes at the time of genetic testing decreased from more than 4 years before 2005 to less than 3 months after 2012 [8].This was due to the introduction of a very simple patient selection and testing strategy; rapid, high-throughput robotic Sanger sequencing analysis of common cause NDM genes by the Exeter laboratory offered free of charge for any patient in the world with diabetes diagnosed under 6 months. Wide spread education and increased recognition of neonatal diabetes as a rare type of diabetes in addition to easily accessible free genetic testing contributed to the significant reduction in time from diabetes diagnosis to a confirmed molecular genetic diagnosis.

The prognosis is linked to the severity of the hyperglycaemia and ketoacidosis and crucially, the speed with which the disease is recognised and treatment implemented.



For peer review only - http://bmjopen.bmj.com/site/about/guidelines.xhtml

A correct genetic diagnosis of neonatal diabetes is important as it determines the most effective form of treatment. For the commonest subtypes of neonatal diabetes due to *KCNJ11* or *ABCC8* mutations most individuals (>90%) can be transferred from insulin injections to sulphonylurea tablets with marked improvement in glycaemic control, with reductions in hypoglycaemia and blood glucose monitoring [9-12]. Twenty percent of mutations in these genes cause neurological impairment and improvements in motor and cognitive function have been reported, with the greatest benefits seen with earlier sulphonylurea treatment, leading to improved glucose control, neurological function and quality of life for the patient and their families [7, 13-15].

In the United Kingdom, all neonates are screened at day 5 of life as part of standard routine postnatal care. Sample collection involves a heel prick blood sample collected on specialist blood spot paper cards. These are analysed by one of a network of NHS Neonatal Screening laboratory for nine rare but serious metabolic diseases. This NHS routine screening service offers the potential for early detection of hyperglycaemia, as insulin deficiency from birth results in a hyperglycaemia that can be detected in the first few days of life [16], thereby facilitating earlier treatment and genetic diagnosis of NDM.

For a specific test to be considered viable for a screening programme, a number of criteria have been suggested [17]. The most widely used are those proposed by Wilson-Jungner, which are designed to appraise the validity of a screening programme and have been adapted for application to screening in the UK [18] The original criteria are:

A. The condition being screened for should be an important health problem

For peer review only

B. The natural history of the condition should be well understood

C. There should be a detectable early stage

D. Treatment at an early stage should be of more benefit than at a later stage

E. A suitable test should be devised for the early stage

F. The test should be acceptable

G. Intervals for repeating the test should be determined

H. Adequate health service provision should be made for the extra clinical workload resulting from screening

I. The risks, both physical and psychological, should be less than the benefits

J. The costs should be balanced against the benefits

Screening for NDM fulfils criteria most criteria, with compelling evidence that early identification of NDM would avoid many of the severe complications and mortality associated with early unrecognised presentation of diabetes. [18]. However the test should also be considered 'acceptable' [18].

Our study aimed to gain understanding of parental experience of the presentation and recognition of neonatal diabetes and their views on the acceptability of adding glucose testing to the national NBS programme.

## Methods

### ***Recruitment:***

Participants were recruited from the NEWBIE 1 study, which comprised families that had genetic testing for neonatal diabetes at the Royal Devon and Exeter NHS Foundation Trust Clinical Laboratories from 2013 – 2020. Ethical approval was granted by the *South West – Cornwall & Plymouth Research Ethics Committee - NREC no: 18/SW/0070).* NEWBIE aimed to establish the diagnostic accuracy of



For peer review only - http://bmjopen.bmj.com/site/about/guidelines.xhtml

new-born screening for neonatal diabetes by assessing glucose at day five of life and the clinicians of 139 children across the UK were contacted to ask permission to approach these families. Agreement was provided by 48 parents who were contacted sequentially and verbal consent was taken by telephone (by KL) and consent forms were posted to potential participants, with 25 subsequently returning written consent. 20/25 had a confirmed genetic diagnosis and therefore comprised the potential cohort for this qualitative study.

As the families had young children and were located across the UK, telephone interviews were considered both convenient and acceptable to parents and were arranged for a time and date that was convenient. 10 interviews were conducted as data saturation was achieved at this point and so no further families were contacted at this stage for this qualitative study.

A qualitative approach, from a social constructivist stance, was used to gain in-depth insight into the presentation and recognition of neonatal diabetes. A social constructivist perspective allows the experience of illness to be viewed from multiple perspectives as social and cultural constructs, with individuals experiences of illness being inextricably linked with their experience of life. Parent's stories or narratives therefore provide a means of contextualising illness experience in a holistic biographical context.The focus of the interviews was on gaining an  understanding of the meaning these events surrounding their child's diagnosis had for the families being studied, and their views on the acceptability of adding glucose to the NBS programme.

**Patient and public involvement**

For peer review only



For peer review only - http://bmjopen.bmj.com/site/about/guidelines.xhtml

Patient and public involvement has been integral throughout the project. The study was initially discussed with 12 members (3 with diabetes) of the NIHR Exeter Clinical Research Facility user group and all agreed the project was feasible and would provide no inconvenience. Three parents of children affected by neonatal diabetes were also contacted and unanimously supported the proposal and two were actively involved in the creating and reviewing patient information. They will also assist us in producing summaries of the project for dissemination to ensure these are clear to non-healthcare professionals and can have maximum impact. The findings of the study have also been shared with the participants as described in the discussion.

In-depth telephone interviews were conducted with 10 parents (8 mothers and 2 fathers) of 14 children with neonatal diabetes, currently aged 1-14 years, median 2 yrs. Eight of the children had transient neonatal diabetes (*KCNJ11* (n=5), *ABCC8* (n=1), *6q24* (n=2) all of whom were in currently remission and 6 had permanent neonatal diabetes: *KCNJ11* (n=4), *INS* (n=1), homozygous *GCK* (n=1). Families were from a variety of ethnic groups: Western European (n=7), Arabic (n=1), Pakistani (n=1), Chinese (n=1) (Table 1).



For peer review only - http://bmjopen.bmj.com/site/about/guidelines.xhtml

Table 1. Characteristics of the families taking part

| Participant | Gene affected | PNDM / TNDM* | Ethnic group | Parent affected (confirmed by genetic analysis) | Currently in remission |
|---|---|---|---|---|---|
| 1 | *KCNJ11* | PNDM | Western European | No | N/A |
| 2 | *6q 24* | TNDM | Western European | No | Yes |
| 3 | *KCNJ11* | TNDM | Pakistani | Parents heterozygous (but unaffected, 1st cousins) | Yes |
| 4 | *ABCC8* | TNDM | Western European | Mother | Yes |
| 5 | *GCK* | PNDM | Arabic | Parents heterozygous | N/A |
| 6 | *6q24* | TNDM | Western European | No | Yes |
| 7 | *KCNJ11* | PNDM | Western European | Mother | N/A |
| 8 | *INS* | PNDM | Western European | Mother | N/A |
| 9 | *KCNJ11* | PNDM | Chinese | No | N/A |
| 10 | *KCNJ11* | TDNM | Western European | Mother | Yes |

For peer review only

### *Data Collection and Analysis*

Telephone interviews were conducted with all participants by an experienced qualitative researcher (MS), who introduced herself and her role within this study. She had previously been in contact with four of the parents in her capacity as senior nurse within the monogenic team when providing clinical advice regarding the management of the neonatal diabetes following a genetic diagnosis. Duration of the interviews was determined by the interviewee's responses, but typically lasted 45-60 minutes. Parents were previously asked to identify and remain in, a private, quiet location for the duration of the interview. The interviews were semi-structured with open questions, utilising a qualitative interview guide which focused on six domains:

1. Experiences of pregnancy / post-delivery period
2. Recognition of diabetes
3. Experience of genetic diagnosis
4. Response to diagnosis and experiences since
5. Awareness of NBS tests
6. Views of adding glucose to the NBS and anything else they felt was important to allow issues that were significant to the families to emerge.

The questions asked and prompts given around these domains were deliberately broad and open ended to allow the participants to describe the issues they considered most important for example *'I'm interested to hear about your response to the diagnosis of neonatal diabetes and your experiences since….'* (see supplementary data). The approach taken was flexible and iterative.

The interviews were audio-recorded to ensure accurate data collection and the interviewer also made notes. Interviews were subsequently transcribed verbatim,



For peer review only - http://bmjopen.bmj.com/site/about/guidelines.xhtml

although names and identifying features were abbreviated to preserve anonymity. All participants were assigned a unique study ID. A thematic content analysis was undertaken using an inductive process, this was conducted manually due to the small number of interviews conducted. Transcripts were read and re-read to enable the researchers to become immersed in the data. Highlighting and colour coding of sections of text was used to identify potential issues and areas of interest. Notes were made on the transcripts to indicate the possible topic areas and these were compared across the different transcripts to generate a list of common themes which were considered and refined into themes of key importance. Interviews were concluded after 10 interviews as data saturation was achieved with no new themes emerging from the data. The transcripts were independently analysed by MS and BK. The initial data analysis was revisited to refine the themes and develop sub-themes with participant quotes identified which supported the themes. Themes were discussed with the research team and discrepancies resolved by consensus. Recurring patterns were identified and relationships between the themes within and across transcripts were recorded. Anonymised interview transcripts were stored on a password protected computer within a secure (swipe card access only) research environment. The results reported in this paper focus specifically on the issues relating to the period prior to / during the diabetes diagnosis and subsequent genetic test and the parents views of NBS for glucose, other issues will be described as appropriate in a separate manuscript.

## Results

3 key themes and 8 sub themes emerged:

***Theme 1: Babies were extremely ill when admitted to hospital***



For peer review only - http://bmjopen.bmj.com/site/about/guidelines.xhtml

*Sub-theme: a) parent's concerns not being taken seriously resulted in delays in admission*

Although symptoms of hyperglycaemia had been reported by parents prior to admission, these had been dismissed by other healthcare professionals and led to delays in diagnosis

*'They had no suspicion at all that something can be wrong…..even GP, doctors look at her 'Oh yeah she's so good, she's fine' .. One day before* (hospital admission) *I said 'She's drinking a lot of milk ..and she's sick a lot now' and* (they said) *'Oh it can happen it's reflux'* (but) *…she was drinking and drinking and drinking' (Participant 1)*

Polydipsia is a recognised sign of hyperglycaemia in children and adults diagnosed with diabetes but that was not recognised in this case.

Other causes of symptoms were assumed and delays in admission resulted

*'She'd not grown since she was 6 weeks old and she kept throwing up her bottles and always demanding more, she was always grumpy ….so we took her to the doctors but there was a sickness bug going round and they said 'Bring her back on Monday' (Participant 4)*

Vomiting is also known to be a feature of diabetic ketoacidosis but again was dismissed as having an alternative cause in both these cases.

*Sub-theme: b) Delays in admission resulted in long stays in intensive care required*

Many of the babies were considered to be extremely ill when they were finally admitted to hospital and families recognised healthcare professionals concerns and babies were frequently taken straight to the neonatal intensive care units

*'They were very worried, she was taken direct to ICU.. it was a nightmare for me (Participant 5).*

Both families and healthcare professionals concern about the wellbeing of the baby was clearly evident.

The uncertainty of the situation added to the families concerns.



For peer review only - http://bmjopen.bmj.com/site/about/guidelines.xhtml

Consequently long stays of several weeks in intensive care were common and were likely to have resulted from delays in diagnosis and degree of hyperglycaemia on admission.

*'He was in hospital for 5 weeks altogether' (Participant 6)*

*'He was in neonatal for about 3 weeks' (Participant 7)*

*Sub-theme: c) The severity of the babies condition on admission increased parental anxiety*

There was clear concern regarding the baby's chances of survival and this impacted emotionally on the families.

*'Stressed, depressed, don't know what was going to happen to him, all a worry… it was frightening and scary and horrible' (Participant 6)*

Delays in recognising the deteriorating health of the neonates and the significance of the symptoms resulted in deferral in admission, increased distress for the parents and prolonged hospital stays.

***Theme 2. Identification of diabetes was not 'standardised' and often perceived to be a 'chance' finding***

*Sub-theme: d) Testing for glucose was often attributed to a single healthcare professional*

A number of the families considered testing of blood glucose was down to 'luck' or the attention of an individual health care professional as opposed to a routinely performed test

*'One of the nurses, she was not asked to do so but, she did a urine sample and they found her sugars were abnormal and that is what made her go into the hospital again so it was lucky for H to find it so early' (Participant 5)*

Participants considered testing for glucose to be random, rather than a systematic investigation prior to ICU admission.

Where an individual healthcare professional was regarded as being the ‘single person’ who had identified the problem, questions were raised about what might have transpired if that healthcare professional was not involved

*‘It was just this one good doctor who came ..and he was marvellous and luckily he come or I don’t know what would have happened …I mean would B have been in a worse state ? …maybe he’d have ‘gone’ for ever (Participant 6)*

Parents were clearly aware their babies could have died if the diagnosis had been further postponed.

*Sub-theme: e) Testing for glucose was advised by the families themselves*

One family, where the baby’s mother was known to have diabetes herself, had insisted their baby’s blood glucose was tested, despite being advised to the contrary

*‘She had an abscess above her bottom ..and she was admitted ..* (my) *Dad said ‘Mum’s diabetic, can you test her* (the baby’s) *blood sugars please?’ and they tried to fob us off they were ‘No, no, no, it won’t be anything like that’ but he was adamant ..and that’s when they did and they* (the blood sugars) *were 18 … she was 2 weeks* (of age)*… it could have completely got missed’ (Participant 8)*

This reinforces the importance of healthcare professionals recognising and responding to families concerns.

*Sub-theme: f) Glucose values on testing were substantially raised*

Extremely high levels of glucose on admission were common, suggesting the diagnosis of diabetes had clearly been delayed

*‘It was 30s, 36 nearly touching 40* (mmol/L) *sometimes’ (Participant 6)*

*‘The blood sugar was extremely high, around 40-50* (mmol/L)*’ (Participant 9)*

For peer review only

*Sub-theme: g) Healthcare professionals did not anticipate diabetes in neonates*

Healthcare professionals were not perceived to expect diabetes to be present at such an early age and parents highlighted the need for education

*'They obviously didn't have a clue about this different type of diabetes ..they kept saying his blood sugars were high and they didn't really expect anything like this …they weren't aware … I do really think they need to know more' (Participant 7)*

Healthcare professional's lack of familiarity with neonatal diabetes added to the anxieties felt by the families

*'The most difficult part for me was the beginning, because literally nobody knew what was going on, it wasn't common, nobody knew what to do and it really scared us' (Participant 2)*

The period prior to diagnosis and the eventual recognition of a rare condition which many healthcare professionals would not be familiar with increased parental anxiety.

*Sub-theme: h) Despite perceiving glucose testing to be a chance finding parents were positive about the care their child received*

Despite a lack of experience with neonatal diabetes, parents praised the care given by the healthcare professionals and recognised this was also a difficult circumstance the teams involved

*'Even though all the medical staff were absolutely amazing I can't fault them …this was a very unknown situation for them… I could see nobody knew what to do really' (Participant 2)*

Due to the rarity of neonatal diabetes it was unsurprising that many healthcare professionals were unfamiliar with this condition however parents were hugely appreciative of the care their child received and this influenced one families view of the NHS



For peer review only - http://bmjopen.bmj.com/site/about/guidelines.xhtml

*'I have been very, very grateful for everything that the diabetes specialist has done for our baby, it actually changed the way I think about the UK health system' (Participant 9)*

Recognition of diabetes and testing of blood glucose was considered by parents to be predominantly a consequence of the location of their baby e.g. in an intensive care setting or down to individual healthcare professionals, as opposed to a routine test undertaken in unwell neonates. Despite healthcare professionals unfamiliarity with neonatal diabetes the families were very grateful for the care their children received.

***Theme 3: Adding glucose to the NBS test was universally considered extremely positive:***

Every single parent interviewed was positive about the possibility of adding a glucose test onto the NBS programme

*'I think it's an absolutely fantastic idea I really, really do it's brilliant .. and I think if it wasn't for me carrying the gene ..they wouldn't probably have got tested, so I think it's an absolutely fantastic idea' (Participant 10)*

Parents could not see any disadvantages to NBS as this would ensure blood glucose was measured close after birth and would prevent missed or delayed diagnosis. They recognised this could avoid symptoms of hyperglycaemia being missed

*'I think it's a good idea, that would have been ideal because we went through weeks of .. the symptoms of diabetes but not picking up on it' (Participant 4)*

and recognised that testing at day 5 of life could prevent deterioration in the condition of babies with diabetes

*'That would be a great idea, I mean before it develops even further.. discovering this at a very early stage is going to be extremely helpful' (Participant 9)*

Potential detection at an early stage was considered very valuable.



For peer review only - http://bmjopen.bmj.com/site/about/guidelines.xhtml

Parents considered this approach would avoid potential problems from a delayed diagnosis

*'She was lucky to be diagnosed at 10 days.. I wonder how she survived. In the future .. it would be much better if they were diagnosed earlier and treated earlier, rather than leaving for later days and leading to damage for the child' (Participant 5)*

They highlighted the possibility of preventing babies becoming seriously ill with all the fear that caused

*'My baby went into coma and nearly died …so if they find it at day 5 …we could have avoided that' (Participant 1)*

Parents recognised that NBS for glucose could prevent babies becoming seriously unwell and dying.

Parents were unanimous in the view that adding glucose to the NBS programme was a positive step which would reduce delayed diagnosis and deterioration in the health of babies with diabetes and prevent other families from going through the trauma they had as a consequence of a delayed diagnosis.

## Discussion

Our study provides the first evidence of parental support for the addition of glucose to the new-born screening programme and in-depth insights into the experiences around diabetes diagnosis during the neonatal period in five different genetic subtypes. The key themes identified in this study highlight the risk of a delayed diagnosis with hyperglycaemic symptoms often missed and diabetes diagnosis often considered by families to be a chance finding. This led to babies being extremely ill when the diagnosis was finally made, resulting in long hospital stays and high levels of distress amongst the families. These delays influenced the families perceptions of



For peer review only - http://bmjopen.bmj.com/site/about/guidelines.xhtml

newborn glucose screening as important to prevent other families going through the same situation with the potential for a much earlier diagnosis.

Recognition and diagnosis of neonatal diabetes is frequently delayed with babies presenting with extreme hyperglycaemia, requiring extended intensive care support. This leads to high NHS care costs and parental distress. Parents considered the addition of a glucose measurement to NBS would reduce delays in the diagnosis of neonatal diabetes and prevent the extent of the suffering they experienced with the deteriorating health of their baby. No parents indicated any concerns or suggested any potential negative consequences of NBS screening for glucose within the interviews.

We have previously shown that glucose levels at day 5 of life in those with neonatal diabetes are already raised and markedly higher than the normal range [16], therefore the introduction of glucose as part of the new-born screening programme could prevent delays in recognition and ensure prompt treatment of neonatal diabetes.

Previous studies have explored attitudes to expanding new-born screening in other conditions but many have focused on the views of clinicians [19] or parents with 'healthy' children [20] with far less attention paid to the views of families living with the conditions [21]. We considered it of paramount importance to gain understanding of those actually experiencing the impact of neonatal diabetes to inform NBS for glucose as it is those who have lived through the experience who are best placed to appreciate the issues involved,

Parental views are recognised to be influenced by contextual factors such as personal experience [21]. In families with haemophilia the vast majority (77%)



For peer review only - http://bmjopen.bmj.com/site/about/guidelines.xhtml

supported new-born screening predominantly because they considered it a means to facilitate early support and treatment, inform decisions about future pregnancies and prevent the ‘diagnostic odyssey’ and difficulties associated with a later diagnosis [22]. Concerns about the detrimental impact, in terms of bonding and parental stress, that an unsought and serious diagnosis can have on early parent-child relationships have been highlighted in cystic fibrosis [23,24] and Spinal Muscular Atrophy (SMA)[25]. Despite the majority (70%) of families and individuals living with SMA being in favour of new-born genetic screening due to delays in diagnosis, those not in favour (whose babies had an extremely curtailed lifespan or a longer period of time before the onset of symptoms), were concerned that new-born genetic screening would prevent families enjoying care-free time with their baby before symptoms emerged [25].

Strengths and Limitations:

A key strength of this study is that participants were from a variety of backgrounds and represented five different genetic causes of neonatal diabetes, with varied treatments and prognosis. Parents were all able to provide ‘insider information’ on their personal experiences of a delayed diagnosis of neonatal diabetes in their families. Copies of the draft paper were sent to the participants to ensure the trustworthiness and credibility of the data and analysis, with an invitation to provide any feedback to the research team. Only four of the participants responded to the draft paper and they indicated they were satisfied with the data and information included and none requested any edits. The number of families interviewed was small and therefore our findings may not represent the views of all families with neonatal diabetes  however data saturation was reached and clear themes identified. However further study including more individuals would be required to support our findings.



For peer review only - http://bmjopen.bmj.com/site/about/guidelines.xhtml

## Conclusion

Our study contributes to an emerging literature that considers the acceptability of new-born screening practices from the vantage point of parents with experience of a child with the condition. Although further study including a larger number of individuals is needed to confirm our findings this study provides the first evidence that adding glucose to the NBS programme is not only acceptable to families but endorsed by them, providing the first evidence of acceptability of testing, fulfilling one of the Wilson-Jungner criteria for adding glucose as part of the NBS programme.


## Acknowledgments

The NIHR Exeter Clinical Research Facility is a partnership between the University of Exeter Medical School College of Medicine and Health, and Royal Devon and Exeter NHS Foundation Trust. This project is supported by the National Institute for Health Research (NIHR) Exeter Clinical Research Facility. The views expressed are those of the author (s) and not necessarily those of the NIHR or the Department of Health and Social Care.

## Funding

MS is a National Institute for Health Research (NIHR) Senior Nurse and Midwife Research Leader. MS and BK are supported by the Exeter NIHR Clinical Research Facility at the University of Exeter. TM is funded by an NIHR HEE Senior Clinical Lecturer Fellowship (ICA-SCL-2016-02-003).


## Author contributions:



For peer review only - http://bmjopen.bmj.com/site/about/guidelines.xhtml

MS, BK and TM conceptualised the study and developed the methodology, MS, BK and KL recruited participants. MS conducted and transcribed the interviews, MS and BK analysed the data. MS prepared the original draft, BK, KL and TM reviewed and edited the drafts and MS revised subsequent editions.

## Conflicts of interest

There are no conflicts of interest to declare

## Data availability statement

Requests for access to data, and to discuss potential collaborations, should be made in writing in the first instance to the Chief Investigator Professor Timothy McDonald timothy.mcdonald@nhs.net

## References:

1. Slingerland AS, Shields BM, Flanagan SE, Bruining GJ, Noordam K, Gach A, et al. Referral rates for diagnostic testing support an incidence of permanent neonatal diabetes in three European countries of at least 1 in 260,000 live births. diabetologia. 2009;52[8]:1683-5.
2. Wiedemann B, Schober E, Waldhoer T, Koehle J, Flanagan SE, Mackay DJ, et al. Incidence of neonatal diabetes in Austria-calculation based on the Austrian Diabetes Register. Pediatr Diabetes. 2010;11[1]:18-23.
3. Iafusco D, Massa O, Pasquino B, Colombo C, Iughetti L, Bizzarri C, et al. Minimal incidence of neonatal/infancy onset diabetes in Italy is 1:90,000 live births. Acta Diabetol. 2012;49[5]:405-8.



For peer review only - http://bmjopen.bmj.com/site/about/guidelines.xhtml

4. Grulich-Henn J, Wagner V, Thon A, et al. Entities and frequency of neonatal diabetes: data from the diabetes documentation and quality management system (DPV). Diabet Med 2010; 27:709.

5. Gloyn AL, Diatloff-Zito C, Edghill EL, Bellanne-Chantelot C, Nivot S, Coutant R, et al. *KCNJ11* activating mutations are associated with developmental delay, epilepsy and neonatal diabetes syndrome and other neurological features. Eur J Hum Genet. 2006;14[7]:824-30.

6. Busiah K, Verkarre V, Cave H, Scharfmann R, Polak M**.** Human pancreas endocrine cell populations and activating *ABCC8* mutations. Horm Res Paediatr. 2014;82[1]:59-64.

7. B Jose UG, T Barrett, AT Hattersley, JJ Milles**.** Glibenclamide controls ketosis-prone diabetes in a 38-year-old woman with Kir6.2 mutation Practical Diabetes International 2009;26[6]:3.

8. De Franco E, Flanagan SE, Houghton JA, Lango Allen H, Mackay DJ, Temple IK, et al. The effect of early, comprehensive genomic testing on clinical care in neonatal diabetes: an international cohort study. Lancet. 2015;386(9997):957-63.

9. Pearson ER, Flechtner I, Njolstad PR, Malecki MT, Flanagan SE, Larkin B, et al. Switching from insulin to oral sulfonylureas in patients with diabetes due to Kir6.2 mutations. N Engl J Med. 2006;355[5]:467-77.

10. Rafiq M, Flanagan SE, Patch AM, Shields BM, Ellard S, Hattersley AT**.** Effective treatment with oral sulfonylureas in patients with diabetes due to sulfonylurea receptor 1 (SUR1) mutations. Diabetes Care. 2008;31[2]:204-9.

11. Zung A, Glaser B, Nimri R, Zadik Z**.** Glibenclamide treatment in permanent neonatal diabetes mellitus due to an activating mutation in Kir6.2. J Clin Endocrinol Metab. 2004;89[11]:5504-7.



For peer review only - http://bmjopen.bmj.com/site/about/guidelines.xhtml

12. Clark RH, McTaggart JS, Webster R, Mannikko R, Iberl M, Sim XL, et al. Muscle dysfunction caused by a KATP channel mutation in neonatal diabetes is neuronal in origin. Science. 2010;329(5990):458-61.

13. Slingerland AS, Hurkx W, Noordam K, Flanagan SE, Jukema JW, Meiners LC, et al. Sulphonylurea therapy improves cognition in a patient with the V59M *KCNJ11* mutation. Diabet Med. 2008;25[3]:277-81.

14. Slingerland AS, Nuboer R, Hadders-Algra M, Hattersley AT, Bruining GJ. Improved motor development and good long-term glycaemic control with sulfonylurea treatment in a patient with the syndrome of intermediate developmental delay, early-onset generalised epilepsy and neonatal diabetes associated with the V59M mutation in the *KCNJ11* gene. Diabetologia. 2006;49[11]:2559-63.

15. Shah RP, Spruyt K, Kragie BC, Greeley SA, Msall ME. Visuomotor performance in *KCNJ11*- related neonatal diabetes is impaired in children with DEND-associated mutations and may be improved by early treatment with sulfonylureas. Diabetes Care. 2012;35[10]:2086-8.

16. McDonald TJ, Besser RE, Perry M, Babiker T, Knight BA, Shepherd MH, Ellard S, Flanagan SE, Hattersley AT. Screening for neonatal diabetes at day 5 of life using dried blood spot glucose measurement. Diabetologia. 2017 Aug 5. doi: 10.1007/s00125-017-4383-3.

17. Public-Health-England. Criteria for appraising the viability, effectiveness and appropriateness of a screening programme. Public Health England publications. 2013

18. Wilson JM, Jungner YG. [Principles and practice of mass screening for disease]. Bol Oficina Sanit Panam. 1968;65[4]:281-393.



For peer review only - http://bmjopen.bmj.com/site/about/guidelines.xhtml

19. Hiraki S, Ormond KE, Kim K, Ross LF. 2006. Attitudes of genetic counselors towards expanding new-born screening and offering predictive genetic testing to children. Am J Med Genet Part A 140A:2312–2319.

20. Hasegawa L.E, Fergus K.A,Ojeda N, Au SM. Parental Attitudes toward Ethical and Social Issues Surrounding the Expansion of New-born Screening Using New Technologies. Public Health Genomics. 2011 Jul; 14(4-5): 298–306.

21. Nicholls SG, Southern KW. Parental Decision-Making and Acceptance of New-born Bloodspot Screening: An Exploratory Study PLoS One. 2013; 8[11]: e79441.

22. Boardman FK, Hale R, Young PJ. New-born screening for haemophilia: The views of families and adults living with haemophilia in the UK. Haemophilia. 2019 Mar; 25[2]: 276–282.

23. Al-Jader LN, Goodchild MC, Ryley HC, Harper PS. Attitudes of parents of cystic fibrosis children towards neonatal screening and antenatal diagnosis. Clinical genetics. 1990. 38 [6] 460-465

24. Grob R. Is my sick child healthy? Is my healthy child sick?: changing parental experiences of cystic fibrosis in the age of expanded new-born screening. Soc Sci Med. 2008 Oct;67[7]:1056-64.

25. Boardman FK, Young PJ, Griffiths FE. New-born screening for spinal muscular atrophy: The views of affected families and adults. Am J Med Genet A. 2017 Jun; 173[6]: 1546–1561.

For peer review only



For peer review only - http://bmjopen.bmj.com/site/about/guidelines.xhtml

**Newbie study: Interview schedule:**

**Introduction**
Re-confirmation of agreement to interview / audio-recording etc

Explanation of purpose of interview: to gain understanding of their experiences and views (no 'right or wrong' answers, all thoughts welcomed), recognition that may bring back some emotional experiences if there's anything they'd rather not talk about that's fine

**Specific topic areas:**

- ***Experiences of pregnancy*** **-** including any awareness of fetal growth problems or known previous experience of genetic insulin production problems
- ***Experiences post-delivery*** **-** any concerns re baby related to glucose / feeding / growth
- ***How the diabetes was detected*** **-** lead up to hospital attendance, symptoms, how child presented, age at onset, severity
- ***Experience of diabetes diagnosis*** **-** reactions to diagnosis, what information was given, when genetic testing was discussed, insulin treatment etc.
- ***Experience / Response to genetic diagnosis -*** how information was explained, what sense they made of this, was this linked to information about treatment change, was there discussion about potential learning difficulties, were they put in touch with other families, what support was provided
- ***Did their child have a heel prick at day 5 of life? -*** What was their understanding / experience of this test, how did they feel about it?
- ***What do they think about a test for glucose being added to that test?*** - can they see any advantages or disadvantages of glucose being tested at day 5, would it have made a difference to them and if so how, what do they feel about glucose being added to that test
- ***What are their experiences since the genetic diagnosis?*** *How is their child doing now, general progress, diabetes treatment, hospital follow*
- ***Anything else they may feel is important.*** *Any topics they'd like to discuss or raise that hasn't been asked about specifically, anything else they'd like to provide further detail of, any advice for healthcare professionals or other families*

**General prompts** (to be used as needed)

- Could you tell me more about that ?
- How did that make you feel ?
- What did you think about that ?

**Close of interview**,

Check how they are feeling having talked through their experience.

Explanation that draft paper will be sent on to them for comments, any additional thoughts or questions to get in touch, thank them for their time and involvement

For peer review only

# Standards for Reporting Qualitative Research (SRQR)*

http://www.equator-network.org/reporting-guidelines/srqr/

## Title and abstract

| | Page/line no(s). |
|---|---|
| **Title** - Concise description of the nature and topic of the study Identifying the study as qualitative or indicating the approach (e.g., ethnography, grounded theory) or data collection methods (e.g., interview, focus group) is recommended | Page 1 (line 4-5) |
| **Abstract** - Summary of key elements of the study using the abstract format of the intended publication; typically includes background, purpose, methods, results, and conclusions | Page 2-3 (lines 24-63) |

## Introduction

| | |
|---|---|
| **Problem formulation** - Description and significance of the problem/phenomenon studied; review of relevant theory and empirical work; problem statement | Page 3-5 (lines 81-126) |
| **Purpose or research question** - Purpose of the study and specific objectives or questions | Page 6 (lines 128-130) |

## Methods

| | |
|---|---|
| **Qualitative approach and research paradigm** - Qualitative approach (e.g., ethnography, grounded theory, case study, phenomenology, narrative research) and guiding theory if appropriate; identifying the research paradigm (e.g., postpositivist, constructivist/ interpretivist) is also recommended; rationale** | Page 6 (lines 149-152) |
| **Researcher characteristics and reflexivity** - Researchers' characteristics that may influence the research, including personal attributes, qualifications/experience, relationship with participants, assumptions, and/or presuppositions; potential or actual interaction between researchers' characteristics and the research questions, approach, methods, results, and/or transferability | Page 9 (lines 179-183) |
| **Context** - Setting/site and salient contextual factors; rationale** | Page 6 (lines 133-147) and Page 9 (lines 179-186) |
| **Sampling strategy** - How and why research participants, documents, or events were selected; criteria for deciding when no further sampling was necessary (e.g., sampling saturation); rationale** | Page 6 (lines 134-141) Page 9 (lines 196-203) |
| **Ethical issues pertaining to human subjects** - Documentation of approval by an appropriate ethics review board and participant consent, or explanation for lack thereof; other confidentiality and data security issues | Page 6 (lines 133-141) and page 9-10 (line 196-210) |



For peer review only - http://bmjopen.bmj.com/site/about/guidelines.xhtml

| | |
|---|---|
| **Data collection methods** - Types of data collected; details of data collection procedures including (as appropriate) start and stop dates of data collection and analysis, iterative process, triangulation of sources/methods, and modification of procedures in response to evolving study findings; rationale** | Page 7 (line 164-170) Page 9 (line 178-208) |
| **Data collection instruments and technologies** - Description of instruments (e.g., interview guides, questionnaires) and devices (e.g., audio recorders) used for data collection; if/how the instrument(s) changed over the course of the study | Page 9 (lines 179-210) |
| **Units of study** - Number and relevant characteristics of participants, documents, or events included in the study; level of participation (could be reported in results) | Page 7 (line 164-170) Page 8 (table 1) |
| **Data processing** - Methods for processing data prior to and during analysis, including transcription, data entry, data management and security, verification of data integrity, data coding, and anonymization/de-identification of excerpts | Page 9 (line 195-210) |
| **Data analysis** - Process by which inferences, themes, etc., were identified and developed, including the researchers involved in data analysis; usually references a specific paradigm or approach; rationale** | Page 9 (line 196-210) |
| **Techniques to enhance trustworthiness** - Techniques to enhance trustworthiness and credibility of data analysis (e.g., member checking, audit trail, triangulation); rationale** | Page 16 (line 356-360) |

**Results/findings**

| | |
|---|---|
| **Synthesis and interpretation** - Main findings (e.g., interpretations, inferences, and themes); might include development of a theory or model, or integration with prior research or theory | Page 10-15 (line 212-327) |
| **Links to empirical data** - Evidence (e.g., quotes, field notes, text excerpts, photographs) to substantiate analytic findings | Page 10-14 (line 212-323) |

**Discussion**

| | |
|---|---|
| **Integration with prior work, implications, transferability, and contribution(s) to the field** - Short summary of main findings; explanation of how findings and conclusions connect to, support, elaborate on, or challenge conclusions of earlier scholarship; discussion of scope of application/generalizability; identification of unique contribution(s) to scholarship in a discipline or field | Page 15 (lines 330-336) |
| **Limitations** - Trustworthiness and limitations of findings | Page 16 (lines 354-362) Page 3 (line 66-78) |

**Other**

| | |
|---|---|
| **Conflicts of interest** - Potential sources of influence or perceived influence on study conduct and conclusions; how these were managed | Page 18 (line 405) |



For peer review only - http://bmjopen.bmj.com/site/about/guidelines.xhtml

| | |
|---|---|
| **Funding** - Sources of funding and other support; role of funders in data collection, interpretation, and reporting | Page 17 (lines 393-396) |

*The authors created the SRQR by searching the literature to identify guidelines, reporting standards, and critical appraisal criteria for qualitative research; reviewing the reference lists of retrieved sources; and contacting experts to gain feedback. The SRQR aims to improve the transparency of all aspects of qualitative research by providing clear standards for reporting qualitative research.

**The rationale should briefly discuss the justification for choosing that theory, approach, method, or technique rather than other options available, the assumptions and limitations implicit in those choices, and how those choices influence study conclusions and transferability. As appropriate, the rationale for several items might be discussed together.

For peer review only

**Reference:**

O'Brien BC, Harris IB, Beckman TJ, Reed DA, Cook DA. **Standards for reporting qualitative research: a synthesis of recommendations.** *Academic Medicine*, Vol. 89, No. 9 / Sept 2014 DOI: 10.1097/ACM.0000000000000388



For peer review only - http://bmjopen.bmj.com/site/about/guidelines.xhtml
